# Dr. Henry Munson Lyman

**Published:** 1905-01

**Authors:** 


Dr. Henry Munson Lyman, of Chicago, died at Evanston, Ill., November 21, 1904, aged 68 years. He was born in the Hawaiian Islands in 1835, and took his academic degree at Williams College in 1858. Soon afterward he entered the Harvard Medical School, graduating, however, from the College of Physicians and Surgeons, New York, in 1861. He then became an interne at Bellevue Hospital, and subsequently served as acting assistant surgeon in the army during 1862 and 1863. He located in Chicago about this time, and later became a teacher at Rush Medical College and in the Woman's Medical School, serving also at different times as a member of the medical staff of several hospitals. He became president of the Association of American Physicians and of the American Neurological Association, having attained high rank as a teacher and practitioner of medicine.

